# Factors influencing utilization of primary health care by elderly internal migrants in China: the role of social contacts

**DOI:** 10.1186/s12889-020-09178-3

**Published:** 2020-07-03

**Authors:** Yanwei Lin, Chengjing Chu, Qin Chen, Junhui Xiao, Chonghua Wan

**Affiliations:** 1 Department of Health Sociology, School of Humanities and Management, Guangdong Medical University, Dongguan, China; 2 School of Humanities and Management, Research Center for Quality of Life and Applied Psychology, Guangdong Medical University, 1#, Xincheng Avenue, Songshanhu District, Dongguan, Guangdong China; 3 Department of Management and Law, School of Humanities and Management, Guangdong Medical University, Dongguan, China

**Keywords:** Social contacts, Primary health care, Elderly, Internal migrants, Factors influencing, China

## Abstract

**Background:**

Utilization of primary health care is an important aspect of elderly internal migrants’ access to screening and preventive services in China. It has been evident that social contacts, such as community engagement, social mobilization, and the ability to communicate were related to health service delivery, but little has been done to explore the relationship between social contacts and utilization of primary health care for this group. This study aimed to explore the factors influencing utilization of primary health care from the perspective of social contacts among elderly internal migrants in China.

**Methods:**

This was a cross-sectional study including 1544 elderly internal migrants in eight cities. Whether these indivdiuals had chosen to participate in the free health checkup organized in the previous year was adopted as an indicator of the utilization of primary health care. The number of local friends and amount of exercise time per day were measured as a proxy for social contacts. Multivariate binary logistic regression was used to investigate the association of social contacts with the likelihood of using primary health care.

**Results:**

55.6% of the respondents were men, and the mean age was 66.34 years (SD, 5.94). 88.6% had received an education of high school or below. 12.9% had no local friends. 5.2% did not exercise. Just 33.1% had participated in a free medical check-up. Social contacts, age, and medical insurance were associated with more use of primary health care among elderly internal migrants in China.

**Conclusion:**

The role of the community in promoting the use of primary health care should be expanded, such as creating community-based campaigns specifically targeting elderly internal migrants or designing social or sports activities tailored to increase the opportunity for contact between local elders and their internal migrant peers.

## Background

Rapid economic development and accelerating urbanization have brought about a drastic redistribution of population in China. The number of internal migrants who moved between regions within the country reached 245 million in 2016, of which 18 million were more than 60 years of age [1]. This elderly migrant population has been growing faster than the working migrant population for 3 consecutive years since 2015, during which time there was a decline in the total number of internal migrants. The number of elderly migrants continues to grow according to the Internal Migrants Dynamic Monitoring Survey, with about one out of every ten migrants being aged 60 years and older in 2016 [2].

Like international migrants, internal migrants are a potentially vulnerable population healthwise due to the longstanding household registration (“hukou”) policy and its dual (rural and urban) governance system in China [3]. Internal migrants do not have the same rights and benefits as the local registered population in a variety of areas, e.g., social services and health care, because they have no “hukou” (are not registered, have no official residency status) in their receiving city [4]. They not only are exposed to a number of health risks before, during, and after migration, but the barriers to getting health services in the receiving cities may hamper their access to necessary health care, especially for the elderly who are more in need of health services [5]. Promoting the health of elderly migrants who need more proactive support is therefore an essential component of public health. Although the theme of working internal migrants (< 60 years old) and health is common in the literature [6] and there have also been some studies focusing on the health of internal migrant women and children [7], elderly internal migrants are rarely noticed.

Studies on international migrants suggest that compared to non-migrants, utilization of healthcare in migrant populations may be unequal because a series of factors regarding the process of migration, such as health status, self-perceived needs, health-seeking behavior, language barriers, and cultural differences, affect migrants’ needs and access [8]. In Europe, studies have found that migrants are inclined to have lower attendance at and referral rates to mammography and cervical cancer screening and less use of consultation by telephone compared to non-migrants [9]. As one of the nation’s most vulnerable minority groups, elderly immigrants in China face more serious health disparities than other groups due to their limited health literacy, with many experiencing significantly bad health outcomes [10]. However, a limited number of studies have examined the utilization of primary health care in China for internal migrants, especially for elderly migrants.

Utilization of primary health care is an important aspect of migrants’ access to healthcare in the form of screening, preventive services, general practitioners, specialists, emergency rooms, and hospitals [11]. Studies in developed countries show immigrants have lower rates of health insurance and less use of health care resources than local populations [9, 12]. Institutional barriers to access include lack of cultural understanding, lack of open access or community clinics, and failure to integrate care with other support organizations [7, 13, 14]. In China, primary health care is mainly provided by community health service centers (stations). With the large number of medical needs caused by huge aging populations, one of the proposed solutions is to set up elderly support systems in the community health centers [15]. For example, the national policy proposes to provide free medical examinations for the elderly aged 60 and over regardless of household registration status as one of the basic public health services [16].

Core functions of primary health care in China include prevention, case detection and management, gatekeeping, referral, and care coordination, etc., which are provided by community health service centers in urban communities [15]. The main determinants impacting their utilization include gender, age, education, work time every day, etc. [17–19]. It is evident that social contacts, such as community engagement, social mobilization, and the ability to communicate are related to health service delivery [20, 21]. But little has been done to explore the relationship between social contacts and utilization of primary health care or the possible mechanisms mediating the relationship, especially for elderly people who are internal migrants [22, 23]. Therefore, our study attempted to explore the influencing factors from the perspective of social contact.

Social contact is a key dimension of poverty and well-being [24] and plays an important role in determining individual health behaviors [25, 26] that should be considered alongside other factors. However, measuring social contact is challenging, since there is no unified definition of what this means [24]. Empirical studies have explored different aspects of social contact, including physical isolation and access to social resources. For example, as a way of drawing attention away from simply counting numbers of social contacts, studies have focused on “people feel that their communities <<DO THIS OR THAT>>” as a proxy for physical isolation [25, 27] or “ties with other people” as a proxy for access to social resources [22, 28]. For migrant elderly people in China, who are often retired, interaction with friends is their main form of social ties, and exercise in the community is the main way they feel engaged with their communities. Referring to the literature and considering the actual situations of the population in question, this study chose the number of local friends and the exercise time per day in the community as indicators of two aspects of social contacts [29].

China has pledged to provide affordable, equitable access to quality basic health care for all its citizens by 2020 [30]. Strengthening the utilization of primary health-care facilities is considered an effective approach to achieve this goal [15, 21]. As the number of elderly internal migrants is increasing, it is necessary to understand their utilization of health services. Elderly internal migrants face more health management problems than local elderly people due to age and migration, policy restrictions, lack of attention, etc. In terms of levels of awareness and utilization of primary health care, elderly migrants are significantly lower than local elderly people [31]. Improving their use of primary health services can contribute to health equity. In this study, we explored how elderly internal migrants use primary health care and what social contact factors influence that use. Our objective is to complement the existing literature by providing further insights into this complex health equity issue.

## Methods

### Data and sampling

Data were derived from the National Internal Migrants Dynamic Monitoring Survey (NIMDMS) that was led and organized by the National Health Commission of the People’s Republic of China (Former National Health and Family Planning Commission) in 2015. The NIMDMS has been conducted every year since 2014 to understand the changing landscape of internal migration, the utilization of public health services, and the management of family planning services. It is a part of the government’s regular data collection, with unified training investigators helping to fill out the questionnaires via face-to-face, home-based interviews. Response rates for the survey were not announced [32]. Ethical approval of this study was exempted by our local IRB because this study only involved analyzing existing public-access data that had been de-identified.

Stratified, multi-stage sampling based on a probability proportionate to size (PPS) sampling method was adopted. The basic sampling frameworks were all migrants’ households, as reported by each village or neighborhood, that did not have “hukou” (registered resident certificate) in the local area and had been living there for more than a month. Townships were randomly selected and followed by village or neighborhoods. In each village or neighborhood, migrants’ households were selected by simple random sampling according to a random number table.

Sampling sites included eight pilot cities (Beijing, Shanghai, Dalian, Wuxi, Hangzhou, Hefei, Guangzhou, and Guiyang). From the perspective of location, Beijing, Shanghai, Hangzhou, Guangzhou, and Wuxi are located in the east, which is more economically developed; Guiyang belongs to the western region, Hefei, the central region, and Dalian, the northeast region. The data was standardized to adjust for bias caused by differences between regions. Distribution of the various sample sizes within these eight cities was made on the basis of maintaining the sample’s representativeness to the whole country and to the provinces. 8000 households were investigated in Beijing and Shanghai, and 2000 in the other cities. In the end, 27,960 migrants’ households participated in the survey, which included 1544 elderly internal migrants over age 60. The Law of the People’s Republic of China on the Protection of the Rights and Interests of Elderly People stipulates that the starting point for the definition of “elderly” is 60 years old; this study adopted the same definition. These 1544 elderly internal migrants served as our target population representing this demographic throughout the country.

### Measurements

#### Variables and utilization of primary health care

Considering that our study focused on elderly internal migrants who had reached the statutory retirement age, we mainly selected indicators of informal (as opposed to formal, or professional) networks to weigh the social contacts of our study participants. In keeping with earlier studies, the number of local friends and the amount of exercise time per day were chosen as indicators of social connection [26, 33]. Meanwhile, age, gender, education, marital status, medical insurance, average monthly household income, and self-perceived health were measured as demographic characteristics. As a national basic public health service project, elderly people over the age of 60 years can receive free medical checkup services in community health service institutions where primary health care is delivered. Whether elderly migrants had chosen to participate in the free medical examination within the past year was adopted as an indicator of their utilization of primary health care.

The study was a cross-sectional study. Cronbach’s alpha of the questionnaire composed of variables in this study was 0.869, and KMO value was 0.634. The questionnaire was well within the internal reliability and the structure validity.

### Statistical analyses

For descriptive analyses, we showed overall demographics, self-reported health characteristics, and social contacts. We calculated average and standard deviations for age and number of local friends, and average monthly household income for interquartile range. Meanwhile, we conducted a classification process and calculated the frequency of each categorical variable thus defined. The chi-square test was used to examine the differences in the utilization of primary health care between subgroups of each categorical variable.

To explore the association between social contacts and utilization of primary health care, multivariate binary logistic regression was performed and odds ratios (OR) and 95% confidence intervals (CIs) were calculated. Three logistic models were adopted: first, the demographic variables entering into the model (Model I), then adding the variables of socioeconomic and physical conditions (Model II), and finally adding variables of social contacts (Model III). In Model III, demographic variables and regional classification as fixed effects were controlled to disaggregate the influence, and other independent variables were subjected to multi-factor analysis using forward stepwise regression (Forward: LR) based on maximum likelihood estimation.

## Results

### Demographic charactoristics, social contacts, utilization of primary health care

The analytical sample included 1544 elderly internal migrants across four districts. Demographic characteristics and social constants are presented in Table 1. 55.6% of our sample was men, and the mean age was 66.34 years (SD, 5.94). 50.2% of the sample were in the range of 60 to 64 years of age. 88.6% of the individuals had received an education of high school or below. 78.2% were married. 74.7% of the respondents were in the eastern region. In terms of medical insurance, 52.5% of them had the New Rural Cooperative Medical Care Insurance (NCMS), which means that more than an half of elderly internal migrants came from rural areas. This is the result of the household registration policy (“hukou”), which states that people with rural household registration (rural “hukou”) can only participate in NCMS in their hometowns (“hukou” location). Other insurances were as follows: the Urban Employee Basic Medical Insurance (UEBMI, 22.8%), the Urban Residents Medical Basic Insurance (URBMI, 9.7%), the Urban and Rural Residents Cooperative Medical Insurance (4.0%), the Free Medical Care (2.1%), and no medical insurance (8.9%). The interquartile range of the migrants’ average monthly household income was 5000–12,000 RMB (or US$784 to US$1881), and 94.7% said they were healthy or basically healthy.

**Table 1:** Demographic and social contacts of internal elderly migrants (*n* = 1544)

	All respondents n (%)	Respondents attending community free medical examinations n (%)
**All**	1544 (100.0)	511 (100.0)
Gender*		
Male	858 (55.6)	304 (59.5)
Female	686 (44.4)	207 (40.5)
Age**		
60–64	775 (50.2)	229 (44.8)
65–69	387 (25.1)	132 (25.8)
70–74	208 (13.5)	75 (14.7)
75–79	125 (8.1)	54 (10.6)
80-	49 (3.2)	21 (4.1)
**Education**		
Primary school or below	683 (44.2)	218 (42.7)
Middle and high schools	685 (44.4)	232 (45.4)
College and above	176 (11.4)	61 (11.9)
**Marital status**		
Married	1208 (78.2)	397 (77.7)
Single	336 (21.8)	114 (22.3)
Regional classification***		
Eastern region	1153 (74.7)	325 (63.6)
Central region	46 (3.0)	14 (2.7)
Western region	169 (10.9)	106 (20.7)
Northeast region	176 (11.4)	66 (12.9)
**Medical insurance**		
None	138 (8.9)	39 (7.6)
NCMS	811 (52.5)	272 (53.2)
Urban and Rural Resident Cooperative Medical Insurance	62 (4.0)	26 (5.1)
URBMI	149 (9.7)	43 (8.4)
UEBMI	352 (22.8)	121 (23.7)
Free Medical Care	32 (2.1)	10 (2.0)
**Self-perceived health**		
Healthy	837 (54.2)	284 (55.6)
Basically healthy	626 (40.5)	201 (39.3)
Unhealthy, but can take care of themselves	69 (4.5)	25 (4.9)
Unhealthy, and cannot take care of themselves	12 (0.8)	1 (0.2)
Number of local friends***		
0	199 (12.9)	23 (4.5)
1–2	248 (16.1)	74 (14.5)
3–4	233 (15.1)	76 (14.9)
5–6	262 (17.0)	95 (18.6)
7–8	91 (5.9)	37 (7.2)
9–10	236 (15.3)	90 (17.6)
Above 10	275 (17.8)	116 (22.7)
Exercise time per day**		
0 min	81 (5.2)	14 (2.7)
Within 30 min	352 (22.8)	116 (22.7)
31–60 min	608 (39.4)	206 (40.3)
61–90 min	61 (4.0)	29 (5.7)
91–120 min	335 (21.7)	108 (21.1)
Over 120 min	106 (6.9)	38 (7.4)

*p < 0.05; ** *p* < 0.01; ***p < 0.001

In terms of social contacts, the average number of local friends was 8.29 (SD, 11.90), but 12.9% had no local friends; 5.2% did not exercise, and 62.2% had exercise time within 60 min per day. In our study, 511 (33.1%) of elderly internal migrants had participated in a free medical check-up offered by community organizations within the previous year. The results of chi-square testing showed significant differences in the use of free medical examination between different subgroups based upon gender (*p* < 0.05), age (*p* < 0.01), region (*p* < 0.001), number of local friends (*p* < 0.001), and exercise time per day (*p* < 0.01). See details in Table 1.

### Association of demographic charactoristics and social contacts with utilization of primary health care

Table 2 displays OR and 95% CIs for the association of demographic charactoristics and social contacts with utilization of primary health care. The collinearity analysis showed that the tolerances of all independent variables were much greater than 0.1, and the variance inflation factors were less than 10, hence multi-collinearity did not exist.

**Table 2:** Results of Binary Logistic Regression of the Relationship between variables and Utilization of Primary Health Care among Internal Elderly Migrants in China

	Model I		Model II		Model III	
	Demographic + region		Model I + socioeconomic and physical conditions		Model II + social integration	
	**OR**	**95% C. I.**	**OR**	**95% C. I.**	**OR**	**95% C. I.**
**Gender**	0.819	0.648–1.034	0.836	0.661–1.058	0.889	0.698–1.134
**Age (years)**						
60–64	1.0	–	1.0	–	1.0	–
65–69	1.259	0.962–1.648	1.257	0.959–1.649	1.403*	1.058–1.861
70–74	1.295	0.924–1.815	1.315	0.935–1.851	1.420	0.999–2.018
75–79	1.665*	1.105–2.510	1.804**	1.182–2.754	2.099***	1.349–3.265
80-	1.617	0.862–3.036	1.782	0.940–3.380	2.171*	1.111–4.243
**Education**						
Primary school or below	10	–	1.0	–	1.0	–
Middle and high schools	1.240	0.970–1.585	1.258	0.982–1.611	1.214	0.925–1.594
College and above	1.334	0.917–1.941	1.363	0.935–1.851	1.245	0.814–1.905
**Marital status**						
Married	1.0	–	1.0	–	1.0	–
Single	0.949	0.707–1.257	0.987	0.734–1.329	1.008	0.740–1.374
**Region classification**						
Eastern region	1.0	–	1.0	–	1.0	–
Central region	1.331	0.693–2.556	1.328	0.690–2.557	1.312	0.662–2.597
Western region	4.525***	3.195–6.409	4.650***	3.268–6.617	4.191***	2.902–6.053
Northeast region	1.500*	1.073–2.097	1.544**	1.101–2.165	1.555*	1.093–2.211
**Average monthly household income**	–	–	0.846*	0.722–0.991	0.885	0.758–1.033
**Self-perceived health**						
Healthy	–	–	1.0	–	1.0	–
Basically healthy	–	–	0.862	0.683–1.089	0.866	0.680–1.103
Unhealthy, but can take care of themselves	–	–	0.890	0.512–1.574	1.011	0.567–1.805
Cannot take care of themselves	–	–	0.104*	0.013–0.861	0.211	0.024–1.865
**Medical insurance**						
None	–	–	–	–	1.0	–
NCMS	–	–	–	–	1.560*	1.016–2.394
Urban and Rural Resident Cooperative Medical Insurance	–	–	–	–	2.370*	1.213–4.632
URBMI	–	–	–	–	1.062	0.609–1.852
UEBMI	–	–	–	–	1.260	0.779–2.037
Free Medical Care	–	–	–	–	0.842	0.344–2.060
**Number of local friends**						
0	–	–	–	–	−1.0	–
1–2	–	–	–	–	2.859***	1.677–4.875
3–4	–	–	–	–	3.318***	1.943–5.665
5–6	–	–	–	–	3.945***	2.341–6.648
7–8	–	–	–	–	4.391***	2.320–8.309
9–10	–	–	–	–	4.377***	2.580–7.426
Above 10	–	–	–	–	4.607***	2.709–7.834
**Exercise time per day**						
0 minites	–	–	–	–	1.0	–
Within 30 min	–	–	–	–	1.661	0.852–3.238
31–60 min	–	–	–	–	1.804	0.943–3.451
61–90 min	–	–	–	–	3.515**	1.538–8.032
91–120 min	–	–	–	–	1.526	0.777–2.997
Over 120 min	–	–	–	–	1.320	0.613–2.840

*p < 0.05; **p < 0.01;***p < 0.001,” -” was reference, *C.I*. Confidence interval

**Fig. 1:**
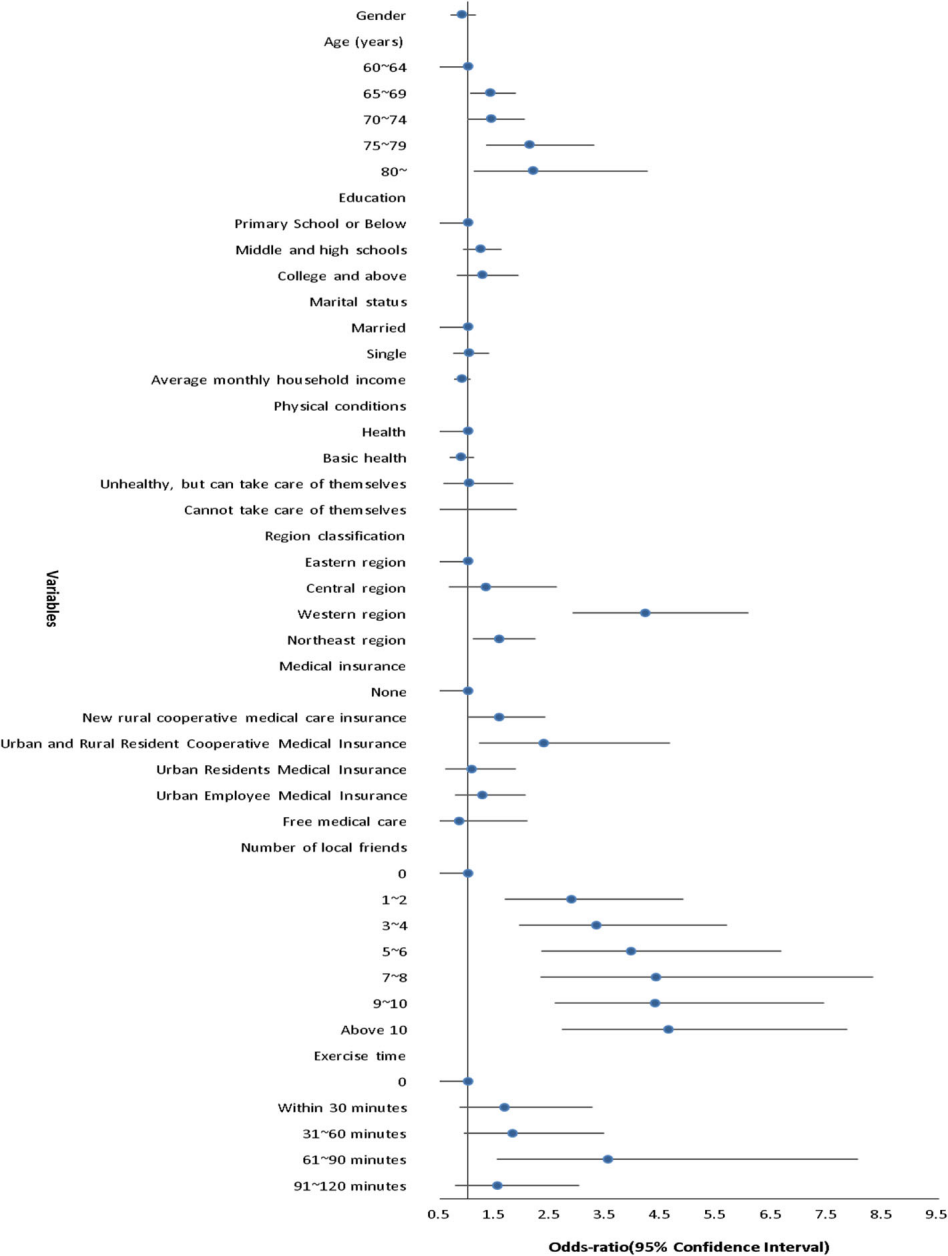
Association between Social contacts and Utilization of Primary Health Care

In the model with demographic and regional variables (Model I), age and region were significant predictors of primary health care use. When socioeconomic and health variables were added, both of them remained significant. Of the other variables, an unexpected finding was that average monthly household income was a risk factor for primary health care use (Model II, OR = 0.846). The social contact variables, when added, all showed positive association with the utilization of primary health care (Model III). The forest map of the OR and 95% confidence values in Model III presents the significance of social contacts and other variables more intuitively (Fig. 1). Other variables that remained significantly associated with the utilization of primary health care were age and region. Comparing the results of Model II and Model III, economic income had an influence in the utilization of primary health care among elderly internal migrants by virtue of the social opportunities it provided for them (Table 2).

Evidence from correlation analysis suggests a significant variation across regions in the utilization of primary health care. Therefore, regional classification and demographic characteristics were controlled in logistic regression Model III as fixed effects. The Hosmer and Lemeshow test indicated that the *P* value (*P* = 0.908) was greater than the inspection level (0.05), which meant that the information in the current data had been completely extracted. The percentage accuracy in classification was 70.4%, suggesting that the means regression model could correctly classify 70.4% of the observations. The odds ratios of the association between social contact and utilization of primary health care are shown in Model III.

As shown in Table 2, Model III, age was significantly associated with the utilization of primary health care. The probability of using primary health care in respondents aged more than 75 years old was more than twice as high as that in respondents aged 60–64 years old (75–79 years: OR = 2.099, 95% CI: 1.349–3.265; ≥ 80 years: OR = 2.171, 95% CI: 1.111–4.243). Similarly, respondents who had social medical insurance care had higher odds of utilizing primary health care compared to those who did not have medical insurance, especially for the people who had the Urban and Rural Residents Cooperative Medical Insurance (OR = 2.370, 95% CI: 1.213–4.632). Respondents in different regions had a different probability of using primary health care, too; elderly internal migrants in the western region even had fourfold higher odds than those in eastern region (OR = 4.191, 95% CI: 2.902–6.053), where the relatively developed cities are located, such as Beijing, Shanghai, Guangzhou, Hangzhou, etc.; those in the northeast region (Dalian) had 55% (OR = 1.555, 95% CI: 1.093–2.211) higher odds than those who lived in the eastern region.

Regardless of the region, associations tended to be stronger for the number of local friends than for the other factors (p < 0.001). Respondents who reported having local friends had a 2.859 ~ 4.607-fold higher odds of utilizing primary health care than those without local friends. In short, the respondents with more local friends were more likely to use primary health care. Respondents who exercised for 61–90 min per day had more than triple (OR = 3.515, 95% CI: 1.538–8.032) the odds of utilizing primary health care than those who did not exercise as much. In a word, elderly internal migrants who had local friends and engaged in 61–90 min of exercise time were more inclined to use primary health care.

## Discussion

The descriptive statistics revealed that most of the elderly internal migrants are aged 60 to 64. There are more men than women, and all tended to have a lower level of education, which is consistent with the characteristics of internal migrants in general. Physical health advantages were also observed, which were similar to those found in previous research [34], but a correlation between physical condition and utilization of primary health care was not found, which contradicts other studies about migrants in general [35]. Two factors may account for this result. First, the elderly who were able to move away from their hometowns were better physically fit generally and not inclined or used to seeking health services in health institutions. Second, for elderly internal migrants as a socially disadvantaged group, we thought social factors that affected their access to primary health care might have played important roles, such as institutional obstacles due to restrictions based upon household registration [35]. Marital status was irrelevant to the utilization of primary health care, which was another inconsistency with previous studies that showed persons aged 65 years or older and living with others were less likely to see a doctor than persons who lived alone [36]. This might be because most of the respondents in our study migrated with their families and the companionship coming from family might have replaced the companionship derived from marriage. Meanwhile, we found that region was related to utilization of primary health care. Whether a person lived in a metropolitan area or a smaller community might lead to different results in utilization of health services, as confirmed by earlier findings of other studies [20]. But how to influence the utilization of health services needs further study. In addition, compared to the 95% coverage of the entire population by just three public insurance plans (NCMS, URBMI, and UEBMI) [37], elderly internal migrants were in an inferior position in terms of their medical insurance.

Compared with the local elderly, migrants had left their hometowns and moved away from their original circle of friends. Notably, we found 12.9% of these elderly had no local friends. Thus, their circles of local friends needed to be rebuilt. In addition, over 60% of internal elderly migrants had not participated in free medical examinations offered in the community, which showed that the obstacles for elderly internal migrants in using primary health care persisted, just as other studies have shown [10, 38]. As the free medical check-ups for the elderly in a community were not restricted by household registration requirements, social contacts have to be considered as a relevant factor.

As we know, the eastern region is more economically developed, as a reference region, but elderly internal migrants who moved there had a lower probability of using primary health care than elderly migrants in other regions. Conversely, the western region, which is least developed, had the highest probability of utilization of primary health care. It was interesting to note that utilization was worse in the regions with higher levels of social and economic development. These results support the idea that there is an inverse association between community social capital and the utilization of primary health care, which has been reported in previously published cross-sectional analysis [20]. Because of this potential bias, we controled for the region while at the same time adjusting demographic, socioeconomic, and health variables. In terms of demographic characteristics, the significant predictors of primary health care use were age and medical insurance, which were consistent with previous research results [39, 40]. We did not find that gender, marriage, education, economic income, or physical health had significant impacts on the utilization of primary health care, contrary to what other studies have discovered [8, 17, 36, 41]. In this case, we believe the discrepancy might be attributed to our target research groups and the type of health services being accessed. First, our subjects were elderly internal migrants who tended to live with their families, because most of the elderly migrants had moved with their children to take care of their grandchildren; therefore, the impacts on the children might be greater than on themselves. Second, we focused on free medical check-up services in the community, so it was reasonable to suggest that in this case economic income was not significant.

As one would expect, besides the influences of the above factors, the number of local friends and amount of exercise time per day were significantly associated with the utilization of primary health care. The elderly migrants with more local friends were more likely to use community health services, which might be due to the information and supports that local friends provided. We noted, as well, that exercise should be encouraged, as exercise time between 60 and 90 min per day was more beneficial for promoting the utilization of primary health care.

### Limitations of the study

This study has merely provided a snapshot of the various factors at work in elderly internal migrants’ use of heath care services. In this approach causality could not be inferred, i.e., the number of local friends and amount of exercise time per day might be a consequence of migrant elders’ attitude to the use of primary health care rather than a reason for primary health care use. Therefore the associations should not be considered to be causal but merely to be correlated. Limitations of this study lay are primarily two. First, the sample size was small, and the number of elderly internal migrants in different sampling areas varied widely, which affected the representativeness of the study. Second, social contact was assessed by the variables of the number of friends and exercise time, while there was no data on the frequency of social contact. Future research would need to examine more extensively both sample size and the measurement of social contact. In addition to number of friends and amount of contact with those friends, future research needs to examine the issue of what those friend networks actually do. We hypothesize that “peer pressure” and group communication about the opportunity for free health services might be the reason elderly migrants would be likely to use or not use primary health care [42]. If a person participated in a free medical examination, it was likely to motivate his/her friends to do the same things. Without that peer influence, the elderly migrants may not be as aware and/or not as motivated to get out and use the services.

## Conclusions

In conclusion, it was important to recognize the role of the influence of social contact on the utilization of primary health care among elderly internal migrants. This study provided a more in-depth examination of the relationship between the two variables and confirmed a positive association. Elderly internal migrants without local friends, medical insurance, and exercise habits should be given more attention. The role of the community in providing that attention should be expanded, such as by creating community-based promotions targeting elderly internal migrants or designing social or sports activities tailored to increase the opportunity for contact between local elders and internal elderly migrants, especially in eastern cities with more developed economies. In view of the growing number of elderly internal migrants, such activities could achieve a significant increase in the welfare of this vulnerable elderly group in China.

## Data Availability

The data used in this paper were provided by the National Health Commission of the People’s Republic of China, which is the top agency governing health issues in China. We had to sign a legally binding agreement with the Commission that we will not share any original data with any third parties. The data, itself, is third party data, and the authors did not produce any of the original data. Interested researchers can apply for access to the data at http://www.moh.gov.cn/ldrks/s7846r/201410/ee63c32ca4b7443faf2feeb14ce88874.shtml and e-mail:ldrkzxsj@163.com, but the final decision is up to the National Health Commission of the People’s Republic of China.

## Abbreviations

OR: Odds ratios
NIMDMS: Internal migrants dynamic monitoring survey
NCMS: New rural cooperative medical care insurance
UEBMI: Urban employee basic medical insurance
URBMI: Urban residents medical basic insurance
